# Transient Activation of Hematopoietic Stem and Progenitor Cells by IFNγ during Acute Bacterial Infection

**DOI:** 10.1371/journal.pone.0028669

**Published:** 2011-12-14

**Authors:** Katherine C. MacNamara, Maura Jones, Olga Martin, Gary M. Winslow

**Affiliations:** 1 Laboratory of Immunology, Division of Infectious Diseases, Wadsworth Center, New York State Department of Health, Albany, New York, United States of America; 2 Department of Biomedical Sciences, University at Albany, State University of New York, Albany, New York, United States of America; University of Ottawa, Canada

## Abstract

How hematopoietic stem cells (HSCs) respond to inflammatory signals during infections is not well understood. Our studies have used a murine model of ehrlichiosis, an emerging tick-born disease, to address how infection impacts hematopoietic function. Infection of C57BL/6 mice with the intracellular bacterium, *Ehrlichia muris*, results in anemia and thrombocytopenia, similar to what is observed in human ehrlichiosis patients. In the mouse, infection promotes myelopoiesis, a process that is critically dependent on interferon gamma (IFNγ) signaling. In the present study, we demonstrate that *E. muris* infection also drives the transient proliferation and expansion of bone marrow Lin-negative Sca-1^+^ cKit^+^ (LSK) cells, a population of progenitor cells that contains HSCs. Expansion of the LSK population in the bone marrow was associated with a loss of dormant, long-term repopulating HSCs, reduced engraftment, and a bias towards myeloid lineage differentiation within that population. The reduced engraftment and myeloid bias of the infection-induced LSK cells was transient, and was most pronounced on day 8 post-infection. The infection-induced changes were accompanied by an expansion of more differentiated multipotent progenitor cells, and required IFNγ signaling. Thus, in response to inflammatory signals elicited during acute infection, HSCs can undergo a rapid, IFNγ-dependent, transient shift from dormancy to activity, ostensibly, to provide the host with additional or better-armed innate cells for host defense. Similar changes in hematopoietic function likely underlie many different infections of public health importance.

## Introduction

Hematopoiesis, the process that supplies the host with innate and adaptive immune cells, is maintained by hematopoietic stem cells (HSCs), which are capable of both self-renewal and differentiation. Under homeostatic conditions, HSCs are thought to be largely quiescent [1], [2], and are commonly referred to as dormant HSCs, or as long-term reconstituting HSCs (LT-HSC), as these progenitor cells have the most robust hematopoietic potential [3]. Although all differentiated blood cells are ultimately derived from HSCs, the daily production of blood and immune cells is provided by more differentiated short-term reconstituting HSCs (ST-HSCs), or multipotent progenitors (MPPs). Much is known regarding HSC potential and differentiation under homeostatic conditions, but how infections can alter the function and phenotype of LT-HSCs is not well understood.

Under steady-state conditions, HSCs and progenitor cells can be identified among the population of cells that lack expression of lineage-specific markers, and express Sca-1 and c-Kit [4]. Alterations in hematopoietic stem and progenitor cell phenotype and function have been observed in bacterial infection models, and during sepsis [5], [6], [7], as evidenced by the apparent expansion of lineage-negative (Lin-neg), Sca-1+ c-Kit+ (LSK) bone marrow cells. Changes in the LSK population have also been observed in mice infected with vaccinia virus and herpes simplex virus [8], [9], and *in vitro* studies have documented an important role for IFNγ in this process [10]. It is likely that, during infection, inflammation acts to modulate hematopoiesis to promote the production of cells better able to respond to and control infection.

Changes in hematopoietic cell activity caused by inflammation or chronic bacterial infection have been associated with a transition of HSCs from dormancy to activity, and this process can be mediated by both type I and type II interferons [7], [11]. It has not been reported whether such a transition is a common feature of HSC biology during acute infections, however. Here, we have used an experimental model of ehrlichiosis to demonstrate that a bacterial infection can cause major, although transient, changes in hematopoietic function that is accompanied by the transition of LT-HSCs and progenitors from dormancy to activity. This process is associated with an IFNγ-dependent expansion of more differentiated hematopoietic progenitor cells. Our data support a model whereby infection-induced IFNγ acts on normally quiescent HSCs to undergo transient activation, in order to promote an expedited innate immune response.

## Results

### Infection-induced LSK cells exhibit altered functional potential

In our previous studies, we demonstrated that *Ehrlichia muris* infection induces major changes to the bone marrow compartment, and enhances myelopoiesis [6], [12]. We initiated the present study to address whether the altered myelopoeisis we had observed was accompanied by changes in bone marrow HSC phenotype and/or function. Such changes were suggested, as, following infection of C57BL/6 mice, we observed an expansion of bone marrow LSK cells (**Fig. S1A**). By day 8 post-infection, the frequency of LSK cells increased by approximately ten-fold, relative to uninfected mice; this increase in frequency corresponded to a 5-fold increase in the number of LSK cells within the bone marrow (**Fig. S1B** and **C**). The apparent expansion of the LSK population was in part due to cell proliferation, as the frequency of LSK cells that had proliferated within a 4-hour interval on day 8 post-infection was increased by three-fold, compared to uninfected mice (**Fig. S1D** and **E**). The frequency of proliferating LSK cells detected on day 16 post-infection was also increased, compared to uninfected mice, but was not as high as that in mice analyzed on day 8 post-infection. The number of BrdU-positive LSK cells among total bone marrow cells was also increased, by over ten-fold, compared to uninfected mice, on both days post-infection (**Fig.S1F**).

**Figure 1:**
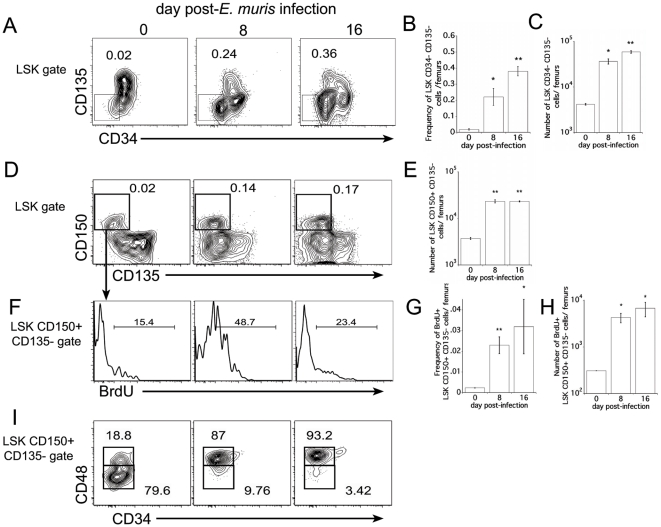
Altered phenotype of LSK cells during bacterial infection. (**A**) Bone marrow LSK cells were monitored for surface expression of CD34 and CD135. The numbers in the plots represent the frequencies of cells within the gated region; the analysis was performed on total bone marrow cells. (**B, C**) The frequency and number of LSK CD34− CD135− cells is shown. (**D**) LSK cells were examined for surface expression of CD150 and CD135. The numbers in the plots represent the frequency of the LSK CD150+ CD135− cells among total bone marrow cells. (**E**) The total number of LSK CD150+ CD135− cells was determined. (**F**) BrdU incorporation was measured in the LSK CD150+ CD135− population 4 hours post-administration. (**G and H**) The frequency of BrdU-positive cells within the LSK CD150+ CD135− population is indicated. (**I**)The LSK CD150+ CD135− cells shown in **D** were analyzed for the expression of CD34 and CD48. The numbers in the plots represent the frequency of CD48-positive or -negative cells among the LSK CD150+ CD135− cells. The flow cytometry plots represent analyses of single mice; the graphs summarize data pooled from at least three mice per group, and are representative of two experiments. A student's t test was performed to evaluate statistically significant differences (* indicates p<0.05, and ** indicates p<0.001). In each case comparisons were made between the uninfected mice and infected mice.

Several published studies have described the phenotypic characterization of HSCs and MPPs, but it is not well understood how the phenotype and function of these populations may change under inflammatory conditions associated with infection. HSCs are found in the fraction of LSK cells that lack surface expression of CD34 and CD135 [13], [14], although many reports have documented significant heterogeneity within this cell population [15], [16], [17]. During *E. muris* infection, the frequency and number of LSK CD34− CD135− cells increased, suggesting that infection promotes the proliferation of true HSCs (**Fig. 1A–C**).

In a model of sepsis, LT-HSCs (i.e., dormant HSCs; characterized as CD150+ CD135− LSK cells) were found to increase in number [5]. We observed a similar increase in the bone marrow LSK CD150+ CD135− population during ehrlichial infection (**Fig. 1D, E**), and these cells were found to incorporate BrdU, suggesting the expansion of the population was, in part, due to proliferation (**Fig. 1F–H**). This was consistent with the observation that LSK CD34− CD135− cells increased during infection. However, given the magnitude of the response, we reasoned that the infection-induced LSK CD150+ CD135− population were not LT-HSCs. To address this in greater detail, we examined the LSK CD150+ CD135− population for its expression of additional markers of hematopoietic differentiation. One such marker, CD48, is a member of the signaling lymphocyte activation marker (SLAM) family, and has been shown to be expressed on multipotent progenitor cells, and is also a marker of actively dividing cells [18], [19]. We observed that, at homeostasis, the LSK CD150+ CD135− population expressed low levels of CD48, but that the infection-induced LSK CD150+ CD135− cells uniformly expressed high amounts of CD48 (**Fig. 1I**), indicating that these latter cells were unlikely true LT-HSCs.

**Figure 2:**
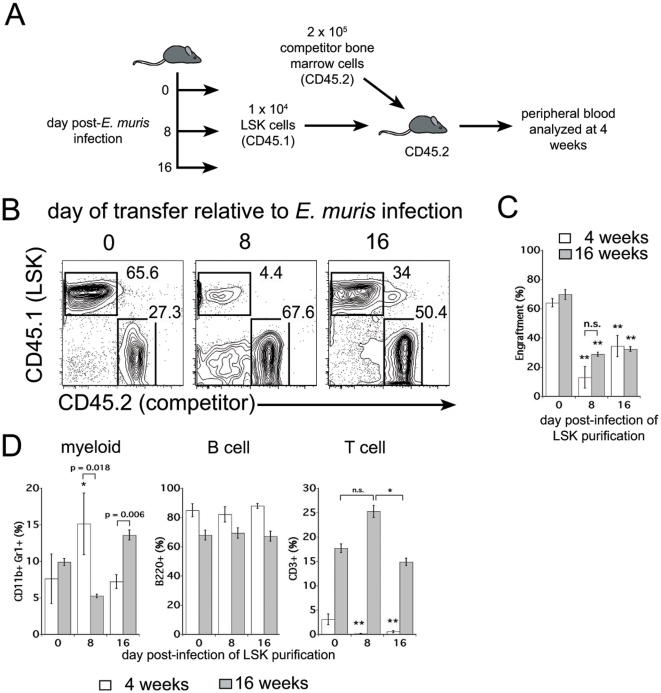
LSK cells purified from infected mice have reduced engraftment potential and are myeloid-biased. (**A**) The schematic illustrates the protocol used for generation of the bone marrow chimeric mice. Bone marrow LSK cells were purified by flow cytometry, and the LSK cells (1×10^4^; CD45.1) were mixed with whole bone marrow (2×10^5^; CD45.2), and were transferred into lethally-irradiated C57BL/6 mice (CD45.2). (**B**) At 4 weeks post-reconstitution, peripheral blood was analyzed for donor cell engraftment. (**C**) The frequencies of LSK-derived cells in the blood of the chimeric mice are shown at 4 weeks and 16 weeks post-reconstitution. (**D**) The frequency of peripheral blood myeloid lineage cells (CD11b+/Gr1+), B cells (B220+), and T cells (CD3+), among LSK donor-derived cells (CD45.1), was determined at 4 weeks and 16 weeks post-reconstitution. A student's t test was performed to evaluate statistically significant differences (* indicates p<0.05, and ** indicates p<0.001). In each case comparisons were made between the mice that received LSK cells from uninfected mice and mice that received LSK cells from infected mice. Significant differences observed between week 4 and 16 post-transplantation are indicated by the brackets.

To further evaluate the function of bone marrow LT-HSCs during infection, we next evaluated the capacity of the infection-induced LSK cells to reconstitute hematopoietic lineages in lethally-irradiated recipient mice. LSK cells were purified from uninfected, or *E. muris*-infected mice, on days 8 and 16 post-infection, and were transferred into irradiated, congenic recipient mice, along with 2×10^5^ radioprotective bone marrow cells (**Fig. 2A**). Peripheral blood from the recipient mice was analyzed for donor cell chimerism 4 weeks post-reconstitution. Whereas approximately 50% of the peripheral blood cells were LSK-donor-derived in mice that received LSK cells from uninfected mice, fewer than 10% of hematopoietic cells were LSK donor-derived in mice that received LSK cells purified from mice on day 8 post-infection (**Fig. 2B**). LSK cells obtained from mice on day 16 post-infection exhibited more efficient engraftment, relative to LSK cells obtained on day 8 post-infection; however, the day 16 LSK cells engrafted less efficiently than the LSK cells purified from uninfected mice (**Fig. 2C**). Thus, during infection, the proliferative status of the LSK population was inversely correlated with its engraftment potential, a finding that is consistent with reports that have demonstrated an inverse relationship between HSC proliferation and function [1]. Thus, despite the apparent increase in phenotypically-identified HSCs, the frequency of functionally-defined HSCs was decreased within the bone marrow LSK population during acute ehrlichial infection.

We next addressed the capacity of the LSK cells from infected mice to contribute to different lineage-positive subsets within the peripheral blood. LSK cells purified on day 8 post-infection gave rise to higher relative frequencies of CD11b/Gr-1-positive myeloid cells than LSK cells purified from either uninfected or day 16-infected donor mice, when examined at 4 weeks post-transplantation (**Fig. 2D**). This myeloid-bias was relatively short-lived, however; by 16 weeks post-reconstitution there were fewer LSK-donor myeloid cells in the periphery of chimeras that received the day 8-infected LSK cells. LSK cells obtained from mice on either day 8 or 16 post-infection also gave rise to very few T cells at 4 weeks post-transplantation; however the frequencies of T cells increased significantly by 16 weeks post-reconstitution and were comparable among all groups at that time.

To verify that lineage potential of the transferred LSK cells in irradiated recipients was due to changes in their differentiation, and not a consequence of secondary bacterial infection in the irradiated recipients, we addressed whether the purified LSK cells contained any bacteria. Bacterial DNA was undetectable in purified LSK cells, but it was possible that small numbers of bacteria were transferred during transplantation. Therefore, we also examined the spleens of irradiated recipient mice for bacterial colonization. The LSK cells did not transmit infectious bacteria, whereas transfer of infected splenocytes resulted in robust bacterial growth in the spleen (**Fig. S2**). Thus, the transient increase in the frequency of myeloid-committed progenitor cells within the LSK population was not a consequence of persistent bacterial infection in transplantation recipients.

### LSK cells from infected mice were less efficient at providing long-term hematopoietic reconstitution

**Figure 3:**
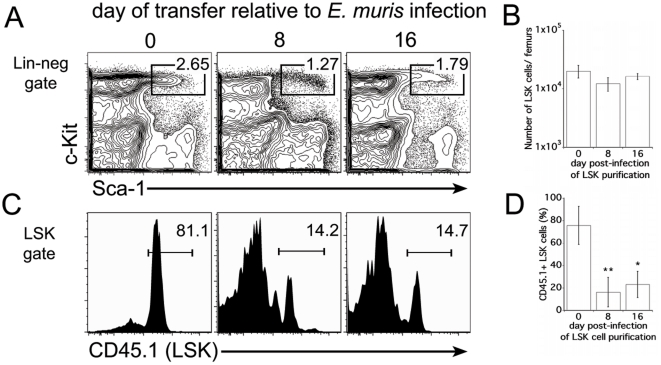
Long-term repopulating activity of LSK cells is diminished during infection. (**A**) The bone marrow of chimeric mice, described in Fig. 2, were analyzed 200 days post-reconstitution. The frequency of the LSK cells, among Lin-negative cells, is shown. (**B**) The average number of LSK cells in the bone marrow harvested from both femurs is shown for each group. (**C**) The LSK population was evaluated for expression of CD45.1. (**D**) The frequency of LSK donor-derived cells is shown. A student's t test was performed to evaluate statistically significant differences (* indicates p<0.05, and ** indicates p<0.001).

To determine the long-term hematopoietic potential of the infection-induced LSK population, radiation-induced chimeric mice that were reconstituted with purified LSK cells, and with radioprotective bone marrow cells, were examined 200 days post-reconstitution. We observed similar frequencies and numbers of LSK cells among the groups of mice that received LSK cells from uninfected, or *E. muris*-infected mice (**Fig. 3A and B**), indicating that the LSK cell reconstitution occurred normally in the chimeric mice. In mice that received LSK cells from uninfected mice, the LSK donor cells composed the majority of the recipient LSK population, likely because there were higher frequencies of HSCs within that donor population. In contrast, chimeric mice that received LSK cells purified from *E. muris*-infected mice contained a much lower percentage of LSK-donor-derived cells; instead, the majority of the LSK population was composed of competitor-derived cells (**Fig. 3C and D**). These data indicate that the infection-induced LSK population contained fewer LT-HSCs with engraftment potential, relative to uninfected mice. Some long-term reconstituting potential, nonetheless, remained within the LSK cell population during infection.

### Infection was associated with an IFNγ-dependent expansion of bone marrow multipotent progenitor cells

IFNγ expression is induced by infection with *E. muris* (**Fig. S3**), and IFNγ signaling is required for protection against *E. muris* infection [6]. Our previous data demonstrated that IFNγ is an important modifier of hematopoiesis during bacterial infection [6], so we also addressed whether the changes in hematopoietic progenitor and stem cells were IFNγ-dependent, by examining these populations in IFNγR-deficient mice during acute infection. The infection-induced expansion of LSK cells was significantly reduced in the IFNγR-deficient mice, as compared to wild-type mice (**Fig. S4**). These data suggest that IFNγ plays a major role in stem cell activation.

**Figure 4:**
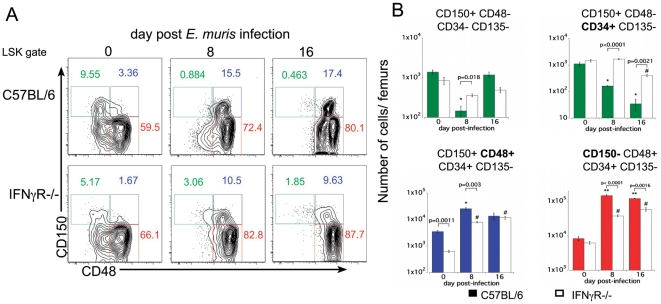
Infection induces an IFNγ-dependent transient depletion of dormant HSCs and a concomitant expansion of MPPs. Hematopoietic progenitor cells were identified in the bone marrow of wild type and IFNγR-deficient mice during acute *E. muris* infection. (**A**) LSK cells were examined for surface expression of CD150 and CD48 on days 8 and 16 post-*E. muris* infection in C57BL/6 and IFNγR-deficient mice. The numbers represent the frequencies of cells, among LSK cells, in each region. (**B**) The LSK CD150+CD48− (highlighted by the green boxes), LSK CD150+CD48+ (blue), and LSK CD150−CD48+ (red) cell populations shown in **A** were evaluated for expression of CD34 and CD135; the number of cells in each population is shown. Data from wild-type (colored histograms) and IFNγR-deficient mice (unshaded histograms) are shown. A student's t test was performed to evaluate statistically significant differences. The numbers of cells in each population in uninfected mice were compared to the equivalent population in infected mice. Statistical significance is indicated for the wild-type mice (* indicates p<0.05, and ** indicates p<0.001), and IFNγR-deficient mice (# indicates p<0.05). Significant differences between wild type and IFNγR-deficient mice are indicated by the brackets.

We characterized the LSK population in greater detail in wild type and IFNγR–deficient mice, using a strategy defined by Wilson et al., who identified LT-HSCs as LSK CD150+ CD48− CD34− CD135− cells [3]. Similar to the findings reported in that study, we found that the LSK CD150+ CD48− population exhibited the lowest proliferation, as measured by BrdU incorporation ([3]; **Fig. S5**). On day 8 post-infection, the number of LSK CD150+ CD48− CD34− CD135− cells was significantly reduced in the bone marrow, relative to the same population in uninfected mice (**Fig. 4A and B**); however, by day 16 post-infection the number of phenotypically described LT-HSCs was equivalent to those observed in uninfected mice. We also characterized other bone marrow progenitor cells, on the basis of CD48 expression. On day 8 post-infection the number of LSK CD150+ CD48− CD34+ CD135− cells was decreased, but the more differentiated LSK CD150+ CD48+ CD34+ CD135− and LSK CD150− CD48+ CD34+ CD135− cells increased in number (**Fig. 4B**). The decrease in the LSK CD150+ CD48− CD34+ CD135− population was not observed in the IFNγR-deficient mice, and the increase in the LSK CD150+ CD48+ and LSK CD150− CD48+ cells was less marked (**Fig. 4B**). These data together suggest that infection-induced loss of phenotypically-defined HSCs was largely IFNγ–dependent, and that this process is accompanied by the expansion of more differentiated progenitor cells.

As Sca-1 expression can be modulated by IFNγ signaling, it was possible that the changes we observed in cell phenotype were a consequence of altered Sca-1 regulation. To address this concern, we examined the Lin- cKit+ population for expression of CD150 and CD48, as these surface antigens are not known to be regulated by IFNγ. The number of Lin-cKit+ cells increased during infection in wild type mice, but decreased in the IFNγR-deficient mice (**Fig. S6A**), thus the population changes were detected using cell surface markers other than Sca-1. Similarly, the Lin− cKit+ CD150+ CD48− population declined, while the CD150+ CD48+ and CD150− CD48+ populations increased in number (**Fig. S6B and C**). These changes were dependent upon IFNγ signaling, and the findings are consistent with a role for IFNγ in driving proliferation of the HSCs and progenitor cells. Thus, these data support our previous study that demonstrated an important role for IFNγ in regulating myelopoiesis during infection, and are consistent with published studies that have described a role for interferons in activating quiescent HSCs [7], [11].

**Figure 5:**
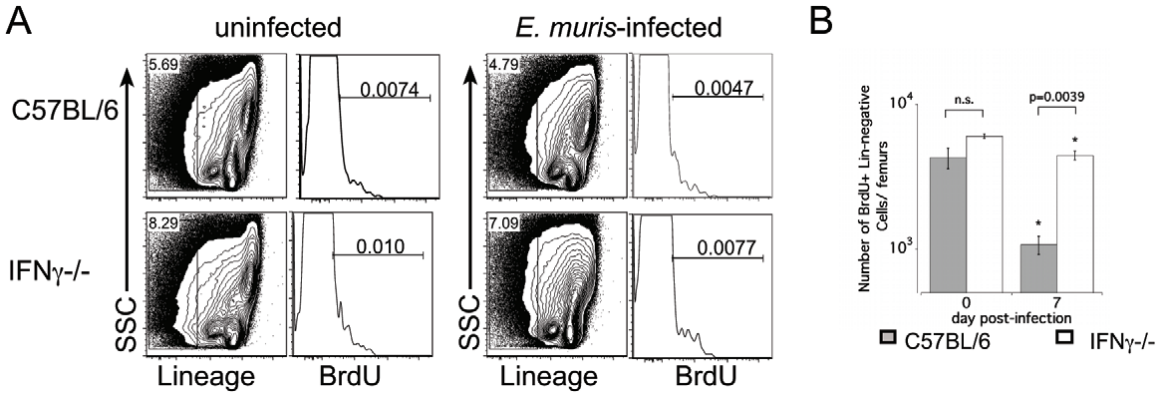
Infection-induced loss of label-retaining cells was dependent on IFNγ. Wild type and IFNγdeficient mice administered BrdU water, *ad libitum* for 12 days; the mice were mock-infected or *E. muris*-infected 165 days following BrdU withdrawal, and analyzed 7 days later. (**A**) BrdU-positive cells within the Lin-negative fraction of bone marrow is displayed for both mock-infected (left) and *E. muris*-infected mice at day 7 post-infection; numbers indicate the frequency of Lin-negative BrdU+ cells among total bone marrow cells. (**B**) The total number of Lin-negative BrdU+ cells in the bone marrow is shown. Statistical significance is indicated between the mock and infected groups (* indicates p<0.05), and significant differences between wild type and IFNγ-deficient mice are indicated by the brackets.

Our data also suggested that IFNγ may act directly on HSCs. To test the hypothesis that IFNγ signaling mediated the activation of the dormant HSC population, we used a label-retaining cell assay to identify dormant HSCs [3], [20]. Wild type and IFNγ-deficient mice were administered BrdU for 12 days, after which time treatment was suspended. Under such experimental conditions, HSCs that incorporated BrdU prior to entrance into a dormant state, retain BrdU for greater than 100 days, and can be detected by flow cytometry [3], [20]. Similar frequencies and numbers of BrdU-positive Lin-negative cells were detected in wild type and IFNγ-deficient mice 165 days after BrdU treatment (**Fig. 5A and B**). When these mice were analyzed 7 days following *E. muris* infection, we observed a five-fold reduction in the number Lin-negative BrdU-positive cells in wild type C57BL/6 mice, but only a small decrease in the number of Lin-negative label-retaining bone marrow cells in infected IFNγ-deficient mice. These data support a role for IFNγ in activating dormant HSCs and/or hematopoietic progenitor cells during acute bacterial infection.

**Figure 6:**
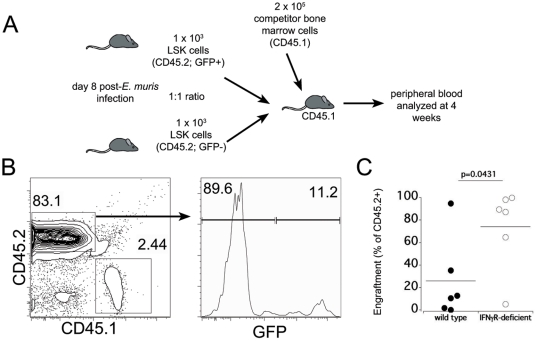
IFNγ signaling reduces engraftment efficiency of LSK cells during infection. (**A**) Equal numbers of LSK cells derived from wild type (EGFP-expressing; CD45.2) and IFNγR-deficient (GFP-neg; CD45.2+) mice on day 8 post-infection were mixed and transplanted in competitive reconstitution experiments. (**B**) Four weeks post-transplant engraftment efficiency was measured in the peripheral blood by donor cell chimerism. The blood from recipient mice (CD45.1+) was analyzed for donor LSK cells (CD45.2+ that were wild type or IFNγR-deficient cells, as determined by expression of GFP. The frequency of GFP+ CD45.2 wild type and GFP− CD45.2 cells is displayed. Statistical significance is indicated between the groups.

As an additional test for our hypothesis that IFNγ signaling acts on HSCs during infection, we performed competitive reconstitution assays, using purified LSK cells obtained from wild type (EGFP-transgenic, GFP+, CD45.2+) and IFNγR-deficient mice (GFP−, CD45.2+) (**Fig. 6A**). The LSK cells were harvested on day 8 post *E. muris* infection, and equal numbers of donor cells were transferred into lethally irradiated congenic CD45.1 mice. At 4 weeks post-transfer, we observed that the LSK cells purified from the IFNγR-deficient mice engrafted much more efficiently than the wild type cells (**Fig. 6B and C**). Thus, we conclude that IFNγ promotes the activation of dormant hematopoietic stem and progenitor cells during *E. muris* infection.

## Discussion

Our study demonstrates that HSCs can undergo a transition from a dormant state to an active state during an intracellular bacterial infection. This conclusion is based on our observations that LSK cells isolated from the bone marrow during infection exhibited increased proliferation, reduced engraftment, and a loss of long-term repopulating potential, all characteristics of activated HSCs. Although it has been demonstrated that HSCs can be activated by IFNγ during chronic mycobacterial infection [7], our study demonstrates that HSC activation can also occur during acute bacterial infection, and, can be reversed quite rapidly. We demonstrate that this transition from dormancy to activation is IFNγ-dependent, supporting the notion that IFNγ acts on HSCs during inflammation. Similar changes in HSCs have been shown to occur following IFNα expression, induced by poly I:C treatment *in vivo* [11]. Thus, an emerging paradigm is that IFNs control the basal state of the dormant HSC population. As most infections trigger the production of IFNs, we postulate that the transient activation of dormant HSCs is a common occurrence.

Among hematopoietic cells, expression of the α chain of the IFNγ receptor is highest in dormant LT-HSCs [7], consistent with the observation that HSC activation by IFNγ occurs via direct signaling on this cell population. Although we have not shown in this study that IFNγ acts directly on HSCs during ehrlichiosis, such a conclusion is strongly supported by our previous data, and other published studies that demonstrated that IFNγ-responsive genes, such as *Adar* and *Irgm1*, are activated in and required by HSCs in response to inflammatory-stress [21], [22]. IFNγ signaling under homeostatic conditions does not appear to be essential, as IFNγ and IFNγR-deficient mice exhibit normal hematopoiesis and bone marrow cellularity.

We hypothesize that, during infection, HSCs transition from dormancy to activity in order to promote the expansion of more differentiated progenitor cells, such as myeloid-biased MPPs. This interpretation is consistent with our previous findings that intracellular ehrlichial infection induced myelopoiesis [6]. In that study we demonstrated that direct IFNγ signaling in promyelocytes resulted in the expression of transcription factors essential for the proper differentiation of granulocytes and monocytes. The changes we have observed are not limited to the ehrlichia. We have observed similar changes to the hematopoietic compartment during influenza and *Mycobacterium tuberculosis* infections (KM and GW, unpublished data), and similar IFNγ–directed alterations in hematopoiesis have been reported during malarial infection [23]. IFNγ signaling has been considered to be detrimental during chronic infection [7], but we propose that IFNγ signaling is essential for activating the hematopoietic response to infection, and for the production of innate immune cells required for combating infection.

Hematopoietic stem and progenitor cells express Toll like receptors (TLRs), and it has been shown that these cells can be directed to differentiate upon direct interaction with TLR ligands [9], [24], [25]. In addition, chronic exposure to LPS, which can stimulate cells via TLR4, results in phenotypic and functional changes within the HSC population [26]. It has been proposed that circulating HSCs and progenitor cells express TLRs so that they can respond directly to pathogens, and differentiate *in situ*, in non-lymphoid tissues [27]. *E. muris* is not known to encode canonical TLR-ligands, so it is likely that IFNγ provides an alternative mechanism to activate HSCs.

Prospective isolation of HSCs based on cell surface marker expression has been an important focus of hematopoiesis research, and has relied on the use of cell surface markers to facilitate the enrichment of cells with the most potent self-renewal and differentiation potential. During infection, expression of inflammatory cytokines, such as IFNγ, can modulate the expression of cell surface proteins used to identify stem and progenitor cells [6]. We have shown that the LSK population, which contains HSCs, undergoes phenotypic and functional changes during ehrlichial infection, similar to findings obtained in studies of viral, parasitic, and other bacterial infections [8], [23], [24], [28]. Initially, we observed that the number of LSK CD34− CD135− cells increased during infection, which suggested that the LSK population contained more HSCs. However, our studies demonstrated that the infection-induced LSK cells exhibited poor engraftment, indicating that the LSK CD34− CD135− cell population contained few LT-HSCs. We showed that the decreased engraftment potential of the LSK population on day 8 post-infection correlated with a decrease in the number of dormant HSCs, where dormant HSCs were characterized as LSK CD150+ CD48− CD135− CD34− cells. By day 16 post-infection, when the dormant HSC population was found to recover, we observed a similar increase in LSK engraftment potential. In a model of cecal ligation-induced bacterial sepsis, LT-HSC cells were shown to expand significantly within the bone marrow [5], findings which are in apparent contradiction to our own. However, in the study by Scumpia *et al.*, the authors defined LT-HSCs as LSK CD150+ CD135− cells. Whereas we, too, found that this population expanded during ehrlichiosis, we observed high expression of CD48 on these cells. Thus, we conclude that the LSK CD150+ CD135− CD48+ cells represent more differentiated MPPs, not LT-HSCs. Thus, additional phenotypic markers may be required to distinguish LT-HSCs from more differentiated progenitor cells under inflammatory conditions.

Our findings, together with the studies from other laboratories, demonstrate that infections can mediate profound changes to HSCs by driving, via interferons, their transient conversion from a state of dormancy to activity. It will be important to gain a better understanding of how IFNγ mediates the activation of dormant HSCs, how IFNγ signaling is regulated in these cells, and how this process impacts host defense during infectious diseases of public health significance.

## Materials and Methods

### Ethics Statement

All animal studies were approved by the Wadsworth Center Animal Care and Use Committee (protocol number: 08-326). The Wadsworth Center is in compliance with all federal and state guidelines for the use of animals in research (assurance number: A3183-01).

### Mice

Mice were obtained from the Jackson Laboratory (Bar Harbor, ME), or were bred in the Animal Care Facility at the Wadsworth Center under microisolater conditions. C57BL/6 mice and the following transgenic and gene-targeted strains were used: IFNγR1-deficient (B6.129S7-*Ifngr1^tm1Agt^*/J; described in this study as IFNγR-deficient), IFNγ-deficient (B6.129S7-*Ifng^tm1Ts^*/J), EGFP-transgenic (C57BL/6-Tg(ACTB-EGFP)1Osb/J), and a CD45 congenic strain (B6.SJL-*Ptprc^a^ Pepc^b^*/BoyJ).

### Bacteria

Mice were infected, via intraperitoneal injection, between 6 and 12 weeks of age, with 50,000 bacteria obtained from infected mouse splenocytes, as previously described [12], [29].

### Flow cytometry and antibodies

Bone marrow mononuclear cells were harvested and prepared as previously described [12]. The antibodies used for flow cytometry included the following: biotin-conjugated lineage markers specific for CD3 (clone 17A2), CD11b (M1/70), Ly-6G (RB6-8C5), Ter119 (Ly-76), CD45R (RA3-6B2), phycoerythrin (PE)-cychrome-7 (Cy7)-conjugated Sca-1 (D7), peridinin chlorophyll protein (PerCP)-Cy5.5-conjugated CD45.2 (104), Alexa700-CD45.2 (104), PE-conjugated-CD45.1, PerCP-Cy5.5-CD150 (TC15-12), allophycocyanin (APC)-conjugated c-Kit (2B8), PE-CD135 (Δ2F10), FITC-conjugated CD34 (RAM34), APC-Cy7-streptavidin, and eFluor450-streptavidin (eBiosciences, SanDiego, CA), as well as Pacific Blue-CD48 (HM48-1; BioLegend, SanDiego, CA). Unstained cells were used as negative controls to establish the flow cytometer voltage settings, and single-color positive controls were used to adjust the instrument compensation. The flow cytometric data were acquired using an LSR II flow cytometer (BD Biosciences), and data analysis was performed using FlowJo software (TreeStar, Ashland, OR).

### BrdU administration and detection

For short-term studies, BrdU (0.8 mg) was administered to mice, via the intraperitoneal route, in a single injection. Four hours post-administration, BrdU incorporation was assessed by intracellular staining, using a FITC-conjugated anti-BrdU monoclonal antibody (PRB-1; BD Biosciences), and cells were analyzed on an LSR II flow cytometer. For label retaining cell assays, mice were placed on BrdU water (0.8 mg/mL plus 10% dextrose) for 12 days. BrdU incorporation was assessed by intracellular staining as described above.

### Flow cytometric cell sorting

LSK cells were identified using FITC-conjugated lineage markers, PE-conjugated Sca-1, and APC-conjugated c-Kit. The LSK population was purified using an Aria II flow cytometer equipped with FACSDiva software (BD Biosciences); the purity of the sorted populations was greater than 90% in all experiments.

### Generation of bone marrow chimeras

C57BL/6 mice (CD45.2) mice were lethally irradiated (950 RADs, administered in 2 doses, 3 hours apart), and were reconstituted with 1×10^4^ purified LSK cells, obtained from mock-infected or *E. muris*-infected CD45.1 congenic mice, and 2×10^5^ bone marrow cells obtained from C57BL/6 mice. For competitive transplantation experiments, LSK cells were purified on day 8 post-*E. muris* infection from EGFP-transgenic C57BL/6 and IFNγR-deficient mice. LSK cells were mixed at a 1∶1 ratio (1×10^3^ cells of each population), and transplanted into lethally radiated CD45.1 recipient mice (with and 2×10^5^ bone marrow cells obtained from congenic CD45.1 mice for radioprotection).

### Statistical analyses

Statistical analyses were performed with a Student's t-test, using Prism GraphPad Software (LaJolla, CA); a P value of<0.05 was considered to be significant.

## Supporting Information

Figure S1 Bacterial infection induces an expansion of bone marrow LSK cells.
(TIF)

Figure S2 Purified LSK cells do not transmit *E. muris* to irradiated transplantation recipients.
(TIF)

Figure S3 IFNγ production is induced by *E. muris* infection.
(TIF)

Figure S4 Expansion of the LSK population in C57BL/6 and IFNγR-deficient mice.
(TIF)

Figure S5 BrdU incorporation in LSK subpopulations.
(TIF)

Figure S6 Analysis of SLAM expression on lineage-negative cKit-positive cells during infection.
(TIF)
