# Supplementary figures and images for: Transient Activation of Hematopoietic Stem and Progenitor Cells by IFNγ during Acute Bacterial Infection
Source: PLoS One. 2011 Dec 14;6(12):e28669. doi: 10.1371/journal.pone.0028669 (PMC3237486; doi:10.1371/journal.pone.0028669)

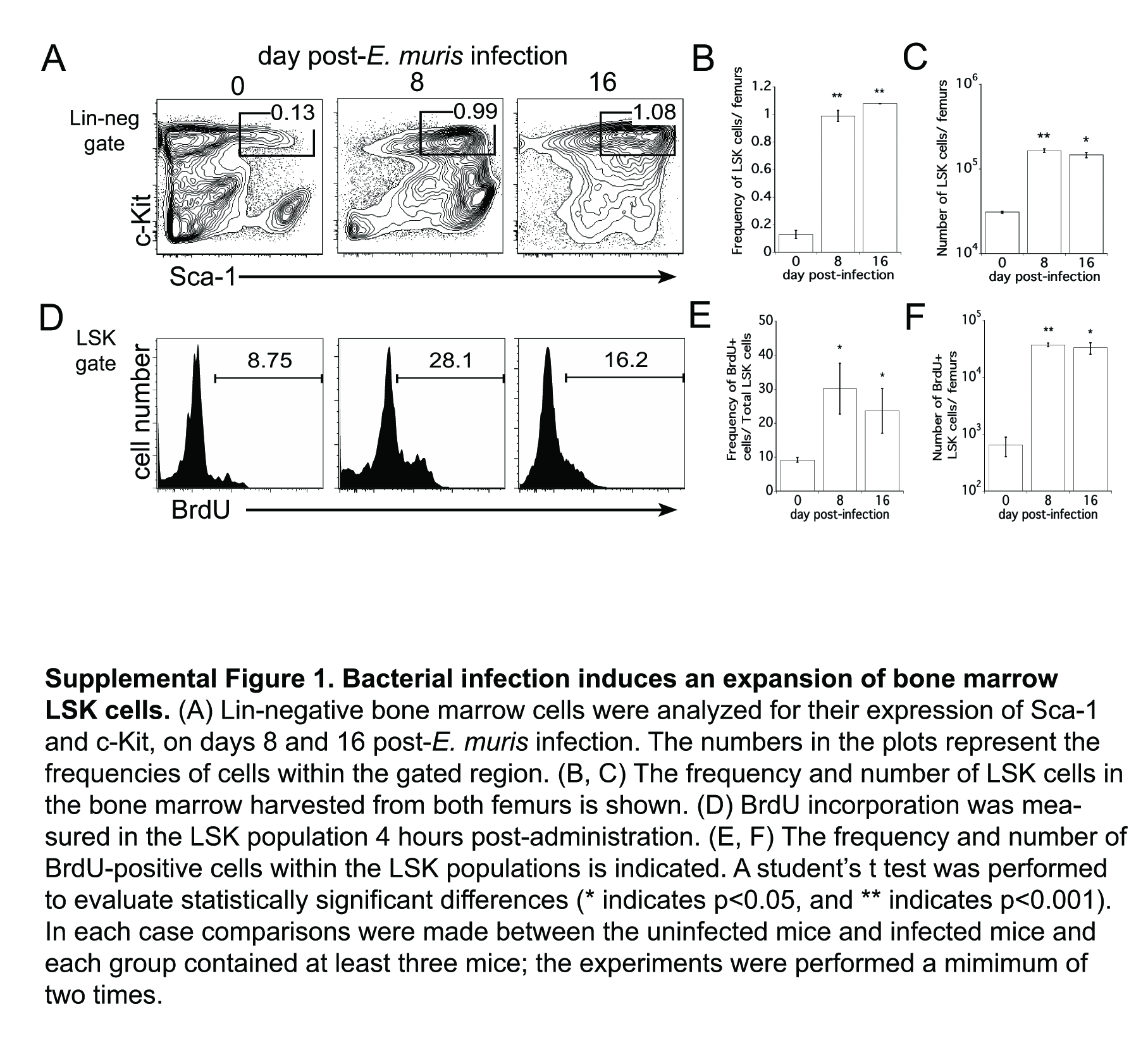

Supplement: Figure S1 — Bacterial infection induces an expansion of bone marrow LSK cells. (TIF) [file pone.0028669.s001.tif]

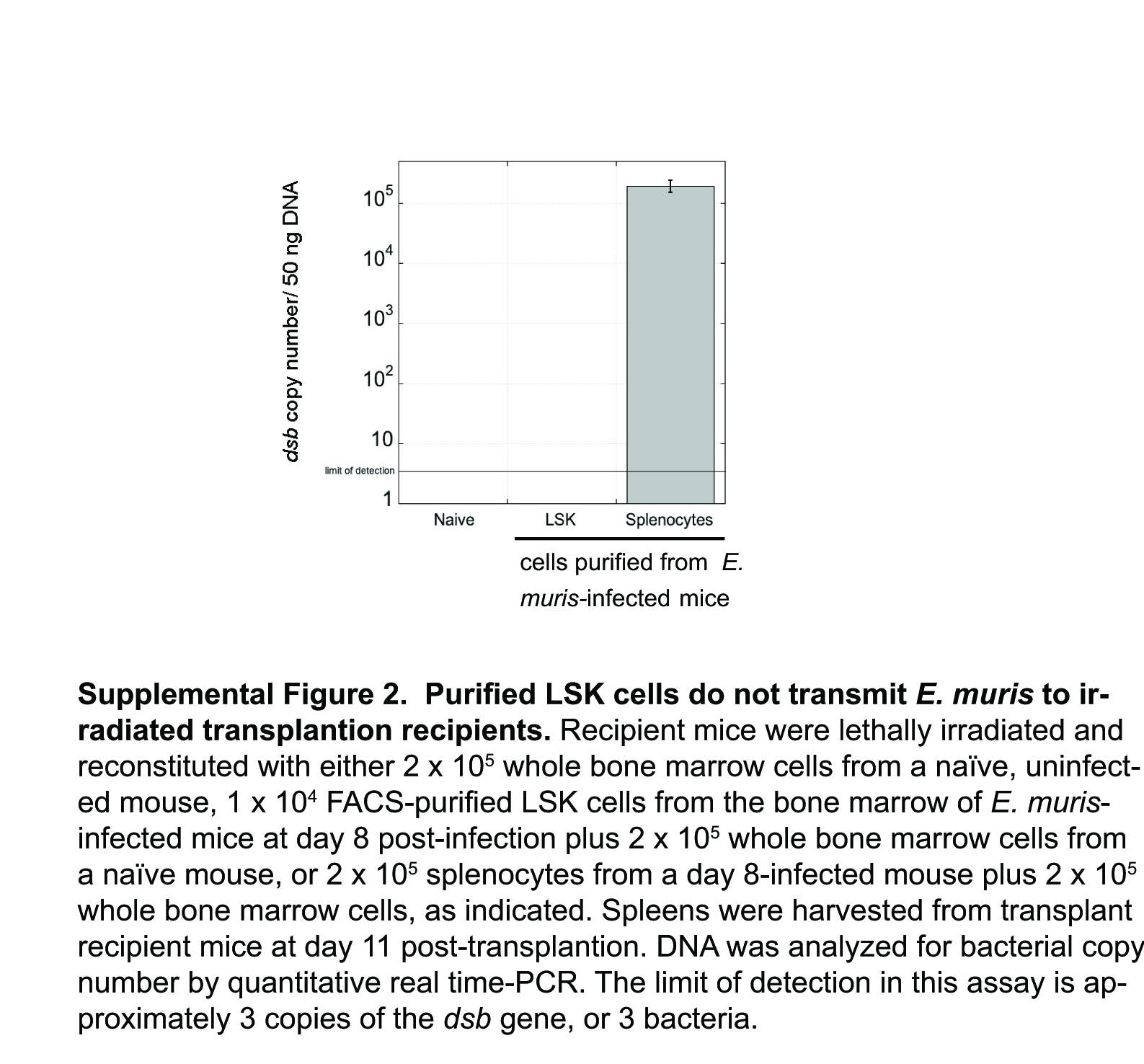

Supplement: Figure S2 — Purified LSK cells do not transmit E. muris to irradiated transplantation recipients. (TIF) [file pone.0028669.s002.tif]

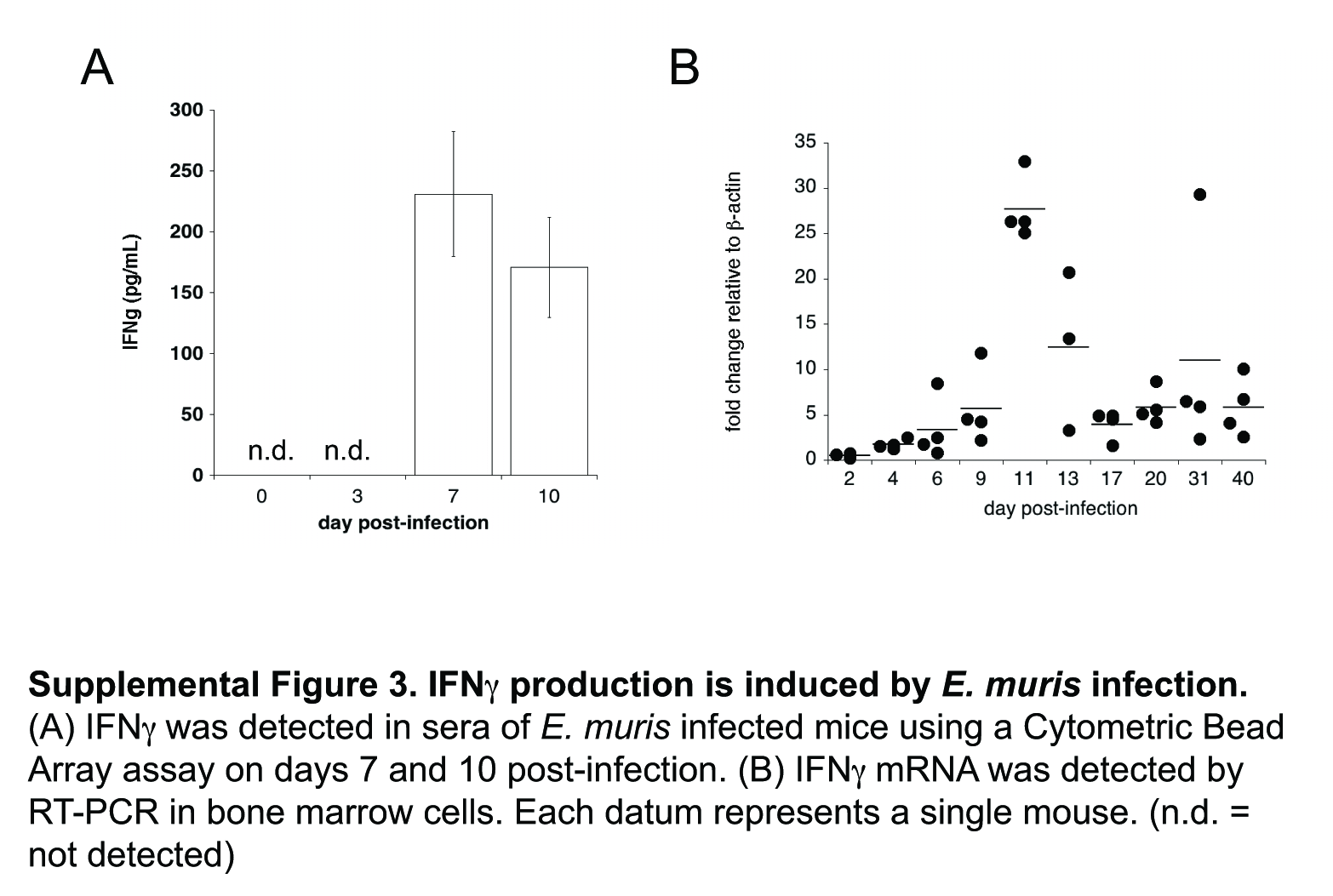

Supplement: Figure S3 — IFNγ production is induced by E. muris infection. (TIF) [file pone.0028669.s003.tif]

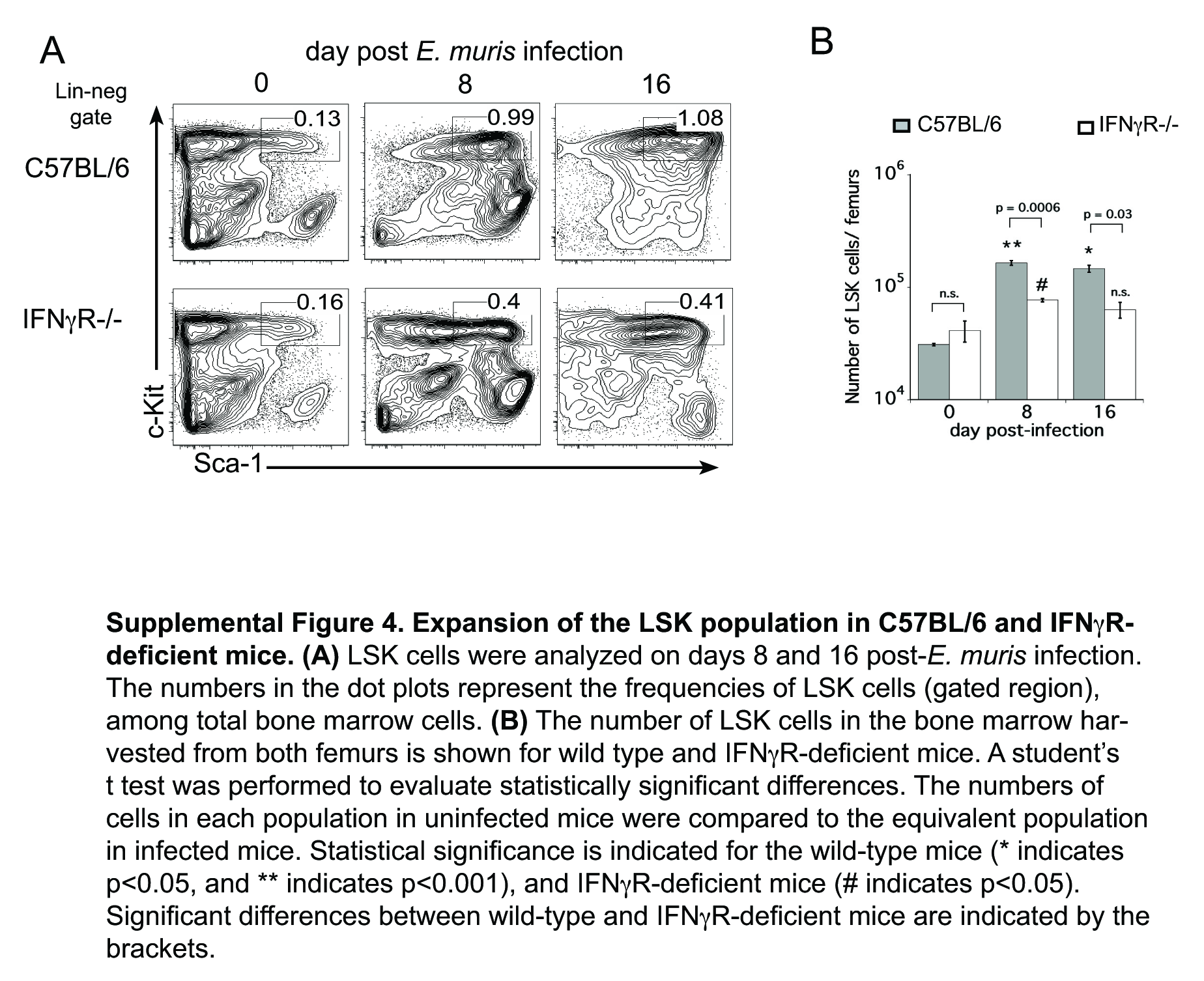

Supplement: Figure S4 — Expansion of the LSK population in C57BL/6 and IFNγR-deficient mice. (TIF) [file pone.0028669.s004.tif]

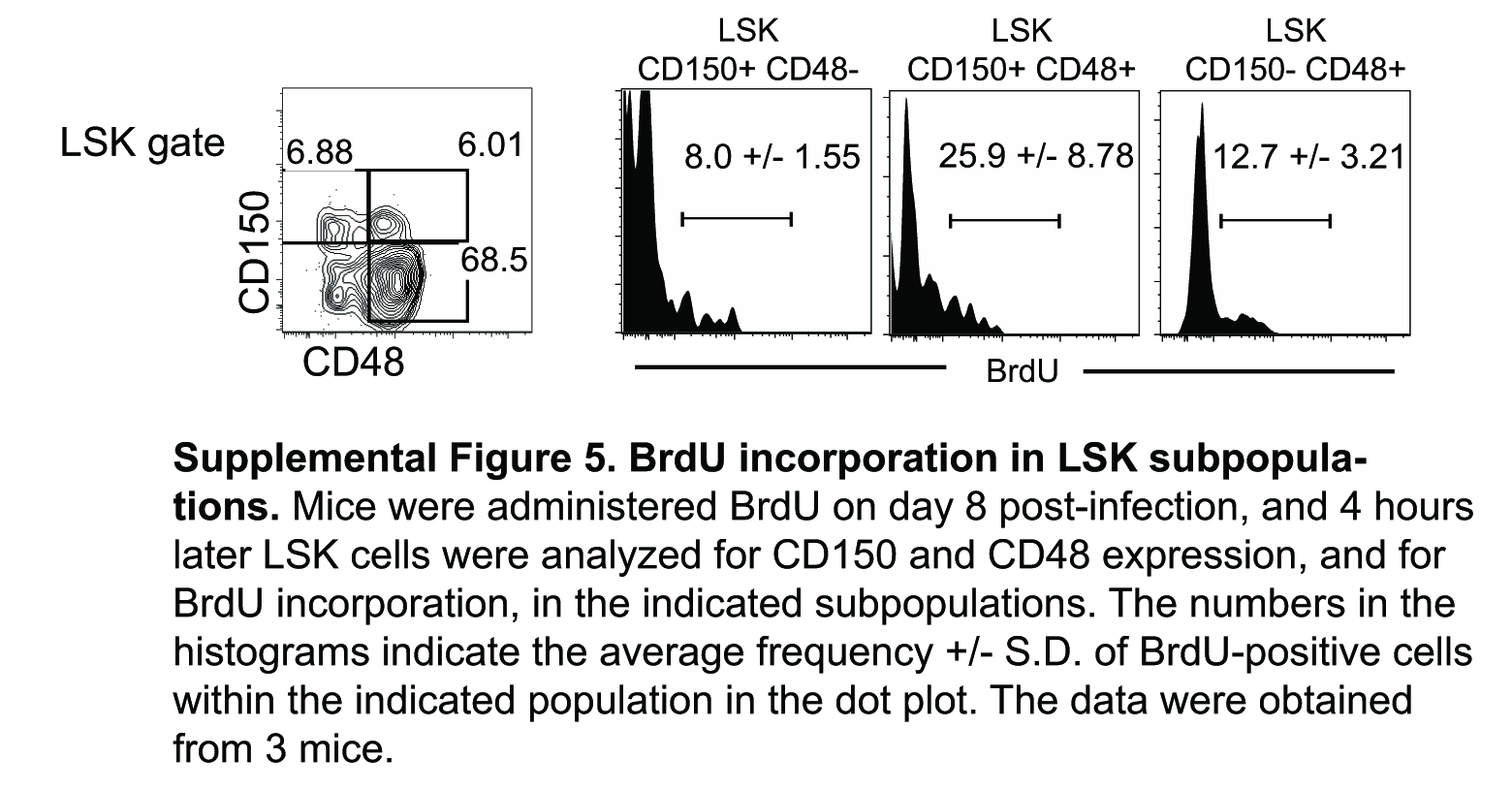

Supplement: Figure S5 — BrdU incorporation in LSK subpopulations. (TIF) [file pone.0028669.s005.tif]

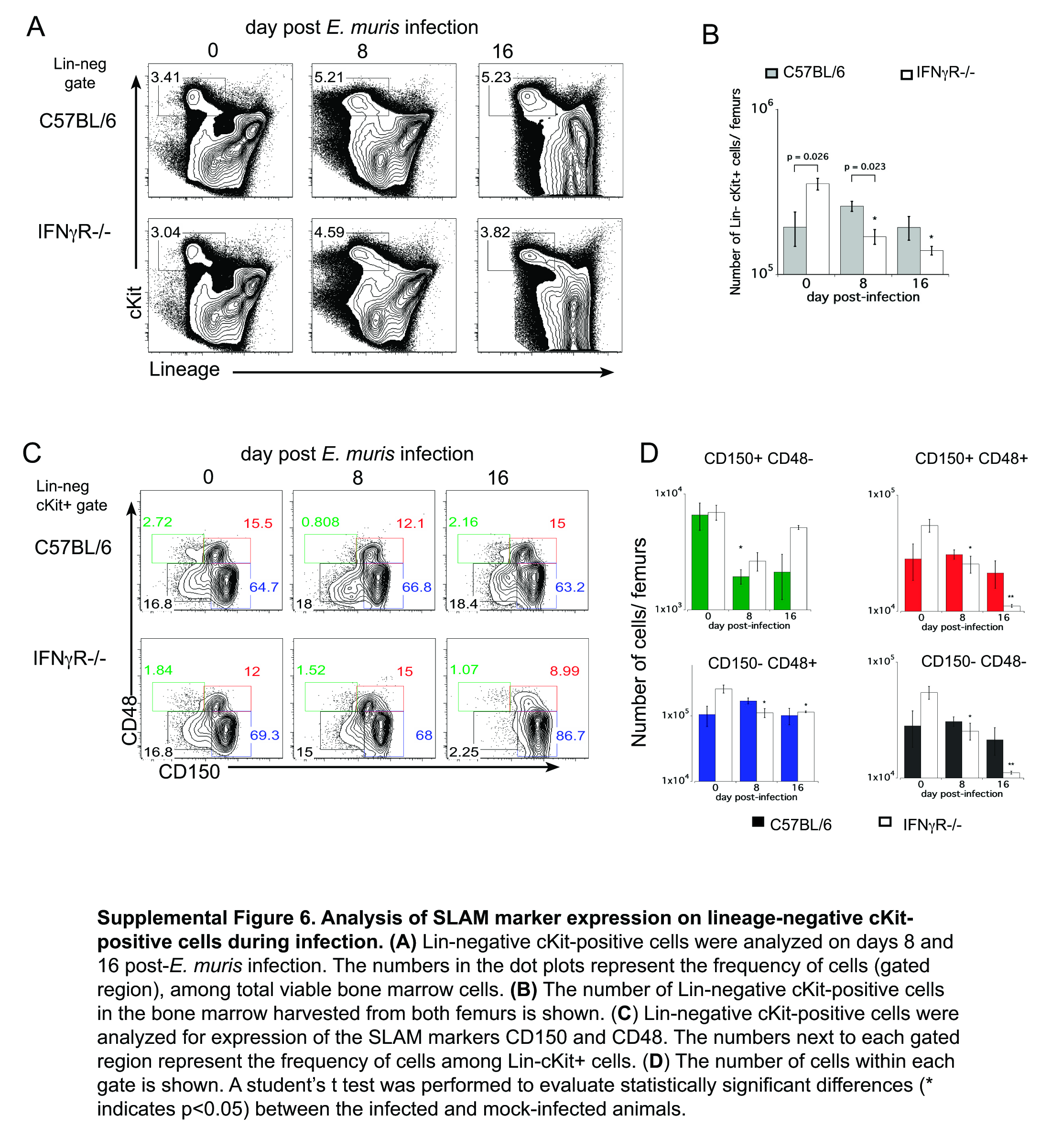

Supplement: Figure S6 — Analysis of SLAM expression on lineage-negative cKit-positive cells during infection. (TIF) [file pone.0028669.s006.tif]
